# *Crocus sativus* L. Extract Containing Polyphenols Modulates Oxidative Stress and Inflammatory Response against Anti-Tuberculosis Drugs-Induced Liver Injury

**DOI:** 10.3390/plants9020167

**Published:** 2020-01-30

**Authors:** Adil Farooq Wali, Jayachithra Ramakrishna Pillai, Yusra Al Dhaheri, Muneeb U. Rehman, Ambreen Shoaib, Omar Sarheed, Salma Jabnoun, Maryam Razmpoor, Saiema Rasool, Bilal Ahmad Paray, Parvaiz Ahmad

**Affiliations:** 1 Department of Pharmaceutical Chemistry, RAKCOPS, RAK Medical and Health Sciences University, Ras Al Khaimah 11172, UAE; jayachithra@rakmhsu.ac.ae (J.R.P.); sarheed@rakmhsu.ac.ae (O.S.); Salma.17903015@rakmhsu.ac.ae (S.J.); Maryam.17903010@rakmhsu.ac.ae (M.R.); 2 Department of Biology, College of Science, United Arab Emirates University, Al Ain 15551, UAE; yusra.aldhaheri@uaeu.ac.ae; 3 Department of Clinical Pharmacy, College of Pharmacy, King Saudi University, Riyadh 11451, Saudi Arabia; 4 Department of Pharmacology, Faculty of Pharmacy, Integral University, Lucknow 226026, India; amber8739@yahoo.com; 5 Forest Biotechnology Lab, Department of Forest Research Management, Faculty of Forestry, University of Putra, Serdang, Selangor 43400, Malaysia; saimu083@gmail.com; 6 Zoology Department, College of Science, King Saudi University, Riyadh 11451, Saudi Arabia; bilalcare@gmail.com; 7 Botany and Microbiology Department, College of Science, King Saudi University, Riyadh 11451, Saudi Arabia; parvaizbot@yahoo.com; 8 Department of Botany, S.P. College, Srinagar, Jammu and Kashmir 190001, India

**Keywords:** anti-tuberculosis, *Crocus sativus*, cytokines, hepato-toxicity, polyphenols, silymarin

## Abstract

The purpose of this study is to analyze the polyphenolic rich extract of *Crocus sativus* L. petals (CSP) in modulating liver oxidative stress and inflammatory response status against rifampicin isoniazid (INH-RIF) drug-induced liver injury. The INH-RIF was administered for 14 days with varying doses in Wistar rats, while silymarin was administered as standard dose. We report the defensive impacts of CSP against INH-RIF induced liver oxidative stress and proinflammatory cytokine. The CSP treatment at both doses significantly controlled all modulating biochemical hepatic injury indicators and resulted in the attenuation of arbitral INH-RIF damage. The components present in CSP identified by LC–ESI-Q-TOF–MS were found to be flavonoids and fatty acids. It can be inferred that CSP possesses a hepatoprotective capacity against INH-RIF-mediated hepatic injury, which may prove to be a medically beneficial natural product for the management of drug-induced liver injury.

## 1. Introduction

Tuberculosis (TB) has been one of the major communicable diseases in the world over the past two decades. Regardless of the development of various new diagnostic tools and medicines in the last 20 years, TB stays a worldwide emergency [1]. World Health Organization reported that in year 2017 around more than 10 million people became sick because of TB and approximately 3 million people die each year [2]. Different medications are used in the treatment of susceptible or mono-resistant tuberculosis such as isoniazid (INH), rifampicin (RIF), pyrazinamide (PZA), ethambutol (EMB), streptomycin, and durazinamide; however, the RIF and INH combinations act as a first-line treatment regimen for 4 to 6 months [3]. Literature survey reported various adverse reactions of antituberculosis (ATC) drugs. One of the adverse reaction of RIF and INH drugs is the development of drug-induced hepatotoxicity (DIH); independently and in combination DIH is amplified in a synergistic manner [4].

Natural products are one of the main source of drugs used by humans worldwide and are currently in huge demand within the context of health care provision and reform [5,6]. Across the globe, various plants are used as traditional medicines for the management of hepatic ailments, currently there are few allopathic medicines available on the market with hepatoprotective activity. Therefore, natural products may offer efficacious, alternative source for the treatment of hepatic disorders [7].

*Crocus sativus* L. (CS) also popularly known as saffron is a flowering plant belonging to the Iridaceae family and cultivated in countries such as Iran, Spain, Italy, etc., [8]. The chemical composition of the CS varies from region to region, seasonal diversification and flora origin [9]. CS contains various secondary metabolites that are responsible for different pharmacological activities. Traditionally CS has been used for various ailments like antihypertensive [10], neuroprotective [11], antitussive [12], aphrodisiac [13], antioxidant [14], antinociceptive [15], anti-inflammatory [16] activities.

Keeping in mind the above considerations, the purpose of the current study is to study the effect of *C. sativus* petals extract (CSP) on the liver oxidative stress and inflammatory response status against INH-RIF-induced liver injury.

## 2. Results

### 2.1. Chemical Profiling of CSP by LC–ESI-Q-TOF–MS

**Figure 1:**
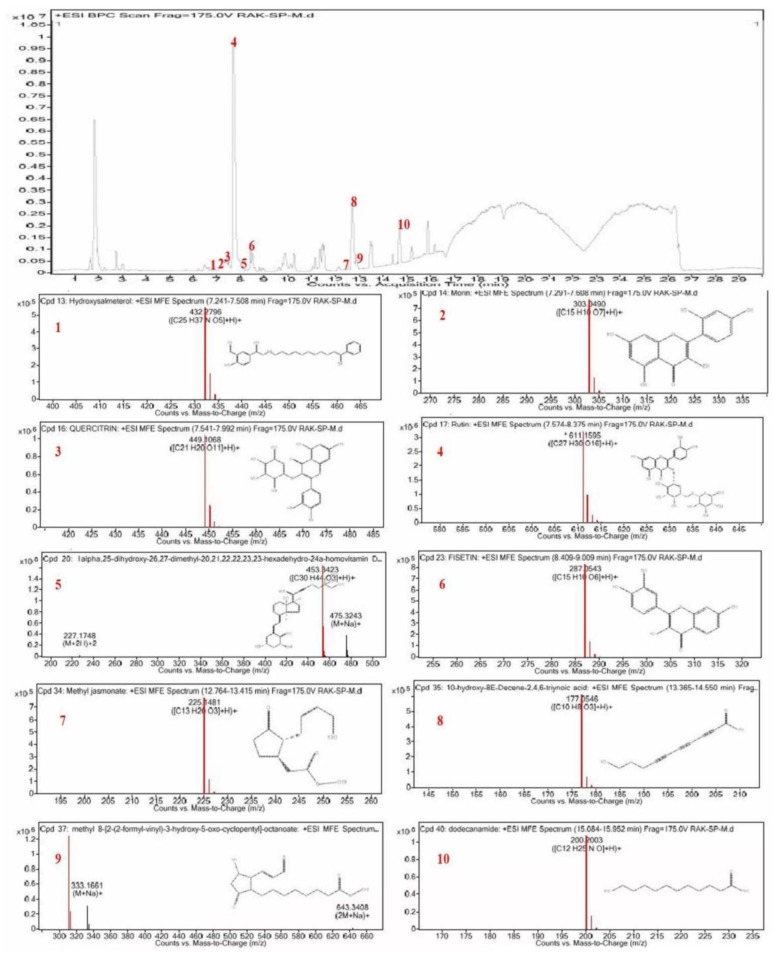
Representative full scan chromatographic profile of CSP and the extracted ion chromatograms.

Polyphenolic rich extract was achieved by ultrasonic homogenizer extraction technique using 90% ethanol as a solvent as described. In the current study, the chemical profiling of CSP polyphenolic rich extract was carried out by means of by LC–ESI-Q-TOF–MS; the molecular mass (m/z) and lambda max (λmax) of the chemical compounds were compared with various database, literature survey, and research articles shown in Table 1. According to the identification data, the chemical compounds identified in CSP polyphenolic rich extract were flavonoids mostly flavonol derivatives along with fatty acids derivatives as shown in Figure 1. The most dominating chemical compound present in the CSP extract was rutin.

### 2.2. Estimation of TPC and TFC

Total phenolic content of CSP estimated by calibration curve with *R*^2^ = 0.992 was 89.63 ± 0.99 mg GAE/g, whereas the total flavonoids content with *R*^2^ = 0.989 was 65 ± 1.09 μg QE/g Table 2.

### 2.3. In Vitro Antioxidant Activity

The IC_50_ value of DPPH and ABTS assays were 99.53 ± 0.63 with *R*^2^ = 0.992 and 116.63 ± 1.93 with *R*^2^ = 0.990 of CSP at a concentration of 150 μg/mL respectively. The results were related with butylated hydroxyanisole as standard sample, which shows an IC_50_ value of 65.46 ± 1.22 with *R*^2^ = 0.901 Table 3.

### 2.4. Elemental Analysis of the CS Petals Using ICP OES

The results of the elemental analysis by ICP OES revealed the presence of 25 micro and macro elements in the CSP. The CSP was found to be very rich in potassium followed by calcium, phosphorous, magnesium respectively as shown in Table 4.

### 2.5. Biochemical Analysis

The findings showed that administration of INH-RIF combination in rats showed a significant increase in the hepatic biomarker concentrations of ALT (*p* < 0.05), AST (*p* < 0.001), and ALP (*p* < 0.01) in rats and a decrease in TP (*p* < 0.05) in rats compared to control group Table 5.

Whereas rats administered with CSP at doses of 100 mg/kg, 200 mg/kg b.wt and silymarin at doses of 10 mg / kg b.wt demonstrated lower regulation of hepatic biomarkers (ALT, AST, and ALP) and higher regulation of TP levels showing hepatoprotective activity of CSP. There was statistically significant reduction of ALT, AST, and ALP levels in rats administrated with 200 mg/kg b.wt CSP as compared with the toxic group.

### 2.6. Estimation of CAT, SOD, and MDA

The oxidative stress efficiency of CSP was measured against hepatotoxicity-stressed INH-RIF. The findings showed that there was a statistically significant decrease in catalase (CAT) and superoxide dismutase (SOD) concentrations in the toxic group and an increase in malondialdehyde (MDA) concentrations compared to the control group.

**Figure 2:**
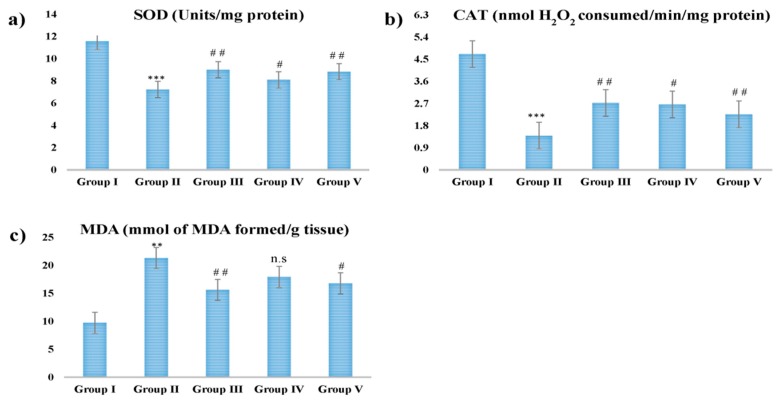
Effect of different doses of CSP and INH-RIF in vivo antioxidant enzymes (**a**) superoxide dismutase (SOD), (**b**) catalase (CAT), and (**c**) malondialdehyde (MDA). Values are mean ± S.E.M; *n* = 6; ** *p* < 0.01, *** *p* < 0.001 vs. control, ^#^ *p* < 0.05, ^##^ *p* < 0.01 vs. group II, n.s stands for not statistically significant.

The rats administrated with 100 mg/kg, 200 mg/kg b.wt, and silymarin at 10 mg/kg b.wt of CSP prevented this induction and CAT, SOD, MDA levels were normalized to their control values. The CSP at the dose of 200 mg/kg b.wt displayed maximum hepatoprotective as shown in Figure 2.

### 2.7. Proinflammatory Cytokine Analysis

**Figure 3:**
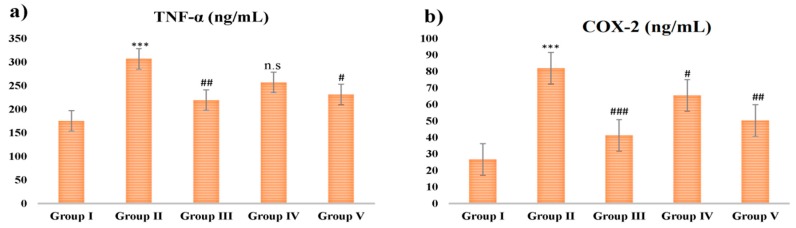
Effect of different doses of CSP on INH-RIF induced proinflammatory cytokines (**a**) TNF-α and (**b**) COX-2. INH-RIF showed steep rise in both the proinflammatory cytokines, administrated with CSP and silymarin decreased TNF-α and COX-2 levels significantly and dose dependently. Values are mean ± S.E.M; *n* = 6; *** *p* < 0.001 vs. control, ^#^ *p* < 0.05, ^##^ *p* < 0.01, ^###^ *p* < 0.001 vs. group II, n.s stands for not statistically significant.

The effect of CSP and INH-RIF treatment on (tumor necrosis factor alpha) TNF-α and cyclooxygenase-2 (COX-2) inflammatory mediators was analyzed. There was an increase in TNF-α and COX-2 levels in toxic group when compared with group I (*p* < 0.001). However, rats of group III, IV, and V administrated with CSP and silymarin showed decreased TNF-α and COX-2 levels significantly and dose dependently (Figure 3).

### 2.8. Histological Evaluation

**Figure 4:**
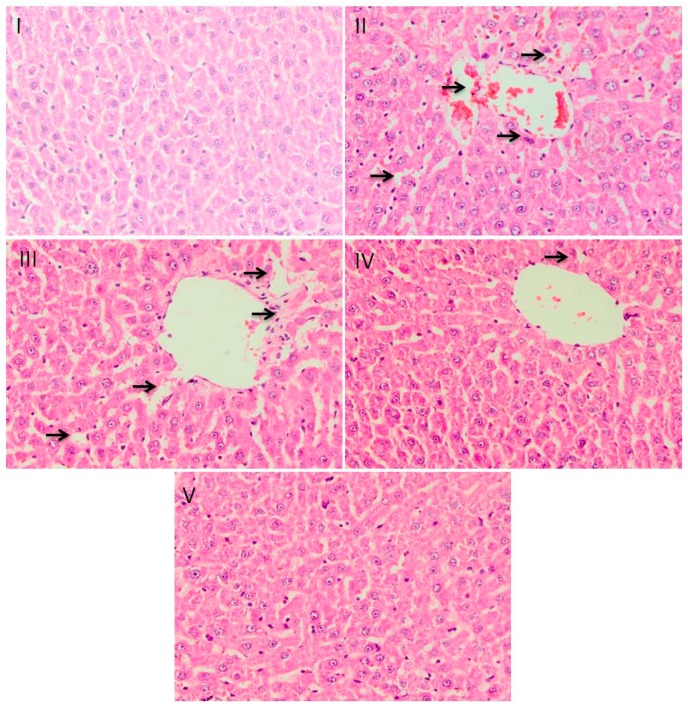
Effect of different doses of CSP on liver histoarchitecture in INH-RIF induced liver injury. Photomicrographs of staining of histological sections of colon depicting different experimental groups, group **I** exhibited the normal integrated cellular architecture. Group-**II** shows extensive disintegration of cells, which is the hallmark of INH-RIF toxicity. In groups **III**, **IV** and **V** CSP treatment showed protection against INH-RIF-induced histopathological damage. magnification: 40×.

Histopathological studies have shown that there is a change in normal liver building design and apparent hepato-cellular necrosis and inflammation in group II compared to group I. Treatment with silymarin in group III prevented all histopathological anomalies caused by INH-RIF. Group III demonstrates highest regeneration of hepatocytes indicating its substantial hepatoprotective activity. Likewise, group V also had shown noticeable recovery, but recovery was lower compared to group III and much more noteworthy compared to group IV (Figure 4).

## 3. Discussion

In the current study, we investigated the effects of CSP on the INH-RIF-induced liver injury in Wistar rats. The phytochemical screening of CSP by LC–ESI-Q-TOF–MS validated that the petals of CS are rich source of flavonoids [17]. Nine different bioactive chemical compounds were present in the CSP belonging mainly to flavonol, a derivative of flavonoids along with some fatty acid derivatives. Fisetin was the predominating component apart from morin, quercetin, and rutin present in this extract; all these compounds can negate the liver toxicity effect caused by INH-RIF via multiple mechanisms [18,19]. Recent studies have shown the health benefits of flavonoids and their potent antioxidant effects [20,21]. Flavonoid compounds are extremely important plant metabolites because of their free radical scavenging ability due to their hydroxyl groups. Therefore, the flavonoid content of plants may directly contribute specifically to their antioxidant and hepatoprotective activity [20,21,22,23]. Our results are compatible with the results of Bathaie and Mousavi [18], which showed that the *C. sativus* stigma possessed higher phenolic, flavonoid content and consequently higher antioxidant activity as compared to *C. sativus* petals [24]. The DPPH and ABTS assay are the most common method used to assess the radical scavenging ability of the different compounds because they have the capacity to give hydrogen free radicals. The mechanism behind the radical scavenging capacity is due to flavonoids and phenolic acids, and these are generally weak in nature, and thus act as competent electrons capable of responding to O2 ш– depending on the substitution within the phenolic ring [25]. Our results are in line with previous literature that CSP was richest in essential and non-essential minerals which are necessary for human wellbeing [26,27]. INH-RIF was selected as a hepatotoxic because previously published studies have shown that INH-RIF induces significant changes in liver cell fortification processes, both enzymatic and non-enzymatic [28,29,30]. During the metabolism of INH by the liver, generation of acetylated metabolites like acetyl hydrazine and isonicotinic acid takes place which are responsible for hepato-cellular injury [31,32,33,34]. Whereas, during the metabolism of RIF, it gets converted to desacetyl rifampicin in liver which may lead to liver toxicity [34,35]. Previous reports suggested that, RIF when co-administrated with INH resulted in increased hepatic oxidative stress, and the synergistic effect of RIF and INH was assumed to be because of CytP_−450_ [36]. The above caused the rise in level of ALT, AST, and ALP due to leakage of enzymes from the liver and reduction in TP level in blood. It also changes the hepatocellular injury which includes hepatic cell augmentation, cholestasis, and alteration of endogenous antioxidants [35]. This study showed, rats treated with CSP at doses of 200 mg/kg b.wt and silymarin at 10 mg/kg b.wt have been shown to more efficiently restore ALT, AST, and ALP enzyme levels to normal. In comparison with our report, Omidi, et al. [36] reported that the *C. sativus* extract decreased the concentrations of hepatic enzyme markers in INH-RIF mediated liver toxicity. CSP exhibits hepatoprotective activity in accordance to the biological activity of flavonoid compounds in the erstwhile reports. Therefore, flavonoids like fisetin, morin, quercetin, and rutin chemical compounds are responsible for antioxidant and hepatoprotective activities of CSP [20,22,23,37,38].

According to Bansal et al. [39] the endogenous antioxidant enzymes present in our body play as a defensive shield against the injury done by free radicals. The SOD enzyme whose purpose appears to reduce the harmful effects due to hydroxyl radical (°OH^−^) by scavenges O^−^_2_ to H_2_O_2_ consequently, reduction of SOD level indicates hepatic injury. Similarly, CAT enzyme degrades H_2_O_2_ into water and oxygen to protect tissue from reactive oxygen species [40,41,42,43]. The risks of hepatotoxicity were raised because of an increase of free radicals and a lack of scavenging capacity of hepatocytes because of increased rates of antioxidant biomarkers [44]. Whereas, increase in the MDA level caused liver injuries because of oxidative stress [45,46]. Therefore, our results reveled that there is significant up regulation of SOD and CAT enzyme levels and decrease in the levels of MDA in the serum of animals. The results reveled hepato-protective activity of CSP at both doses.

The combination of INH-RIF metabolites stimulates Kuffer cells which cause activation of proinflammatory cytokines receptors such as TNF-α and COX-2, which are in turn involved in apoptosis and inflammation of the hepatocytes [47,48,49,50,51]. Our findings show that the concentrations of TNF-α and COX-2 in the INH-RIF-treated animals significantly increased as compared to the controlled animals. Nonetheless, following the administration of CSP and silymarin to INH-RIF-treated animals, the levels of TNF-α and COX-2 decreased significantly. Our results further proposed that hepatoprotective activity effects of CSP at 100 and 200 mg/kg b.wt were due to its potential to decrease the levels of both proinflammatory cytokines markers.

Histopathology has also shown that the treatment of CSP has provided significant immunity against hepatic damage. Metabolism of INH-RIF occurs largely in the liver, which is responsible for the susceptibility of the organ to metabolic-dependent, drug-induced damage [52]. These metabolites may be electrophilic compounds or free radicals that experience number of chemical reactions, like those of depletion of reduced glutathione; covalent linking to proteins, lipids, or nucleic acids; or causing lipid peroxidation [53]. CSP and its flavonoids demonstrated significant prophylactic impact as a result of antioxidant activity and is apparently seen in histological findings of liver sections with distinct hepatocytes, sinusoidal spaces, central veins, and mild degrees of fatty change, necrosis infiltration almost comparable to the group III group. These findings are in accordance with the earlier reports stating the role of flavonoids against hepatotoxicity and oxidative stress [25,54]. Previous studies have also established that flavonoids reverse INH-RIF-induced fibrosis and necrosis [55].

The hepatic biochemical markers, the endogenous antioxidant enzymes, proinflammatory cytokines markers the histological evaluation revealed that the CSP mostly at the dose of 200 mg/kg b.wt significantly attenuated the liver injury, which supports with the results of lower hepatic biochemical markers.

## 4. Materials and Methods

### 4.1. Plant Material and Extraction

*C. sativus* L. petals (CSP) were collected from Pampore (34.02° N 74.93° E.), south of Kashmir, India. After the collection, CS petals were dried in the shade and coarsely powdered. Then this coarse powder was transferred into a 500 mL reagent bottles (Borosil^®^) and extracted with 90% ethanol using the ultrasonic homogenizer for 60 min at 35 °C, 15 kHz (BioLogics, Inc. 300VT). The extract was filtered with Whatman No. 1 filter paper (Supertex, grade 1), evaporated in vacuo (Buchi Rotavapor^®^ R-210) and lyophilized using BenchTop Pro with Omnitronics^TM^ freeze dryer. The CSP was then placed in the desiccator for further testing.

### 4.2. Chemical Profiling of CSP by LC–ESI-Q-TOF–MS

The phytochemical testing of plant polyphenols was carried out using an LC-MS instrument consisting of the Agilent 6200 series TOF/6500 series (Agilent ^®^ Technologies, Palo Alto, CA, USA) connected to the Agilent HPLC 1290 Infinity Binary Pump (Agilent ^®^ Technologies, Palo Alto, CA, USA) with an ESI interface. Zorbax Eclipse C18 column (5 μm, 150 mm × 2.1 mm) was used for chromatographic separation at a flow rate of 0.2 mL/min with two separate mobile phases. Mobile phase (A) water and mobile phase (B) 90 per cent acetonitrile (0.1 per cent formic acid) with gradient system Table 6. The temperature of the column was maintained at 40 °C and injection volume was 3 μL with total run time of 30 min. The LC-MS operating parameters are as follows: the spectra were obtained in ESI+ and ESI− modes in the range of *m/z* = 130–1000, gas temperature 250 °C, gas flow 13 L/min, nebulizer 35 psig, sheath gas temperature 300 °C, Sheath Gas Flow 11 capillary voltage 3.0 kV, and Fragmentor 125 V. All spectral data were collected using a PDA detector. All information, acquisitions, and evaluation were regulated using the Agilent Mass Hunter Software version and the Agilent Database Library was used to verify the compounds.

### 4.3. Estimation of Total Phenolic Content (TPC)

Folin-Ciocalteus assay was used to assess the total phenolic content of the CSP [51].

### 4.4. Estimation Total Flavonoid Content (TFC)

The colorimetric method was used to determine the total phenolic content of the CSP [56].

### 4.5. In-Vitro Antioxidant Activity

The antioxidant activity of CSP were determined by DPPH [24] and ABTS [57] assay.

### 4.6. Elemental Analysis of the CS Petals Using ICP OES

Elements macro and micro were determined by using Spectro genesis ICP OES analyzer (SPECTRO Analytical Instruments, Germany). Calibration curves of standard solutions were used to evaluate each element.

### 4.7. Induction of Hepatotoxicity in Wistar Rats

Four to five-week-old male Wistar rats (165–190 gm) were used in the current study. All the experimental animals were kept under controlled environment and acclimatized for a week, fed *ad libitum* food and water. The investigations were conducted as per the “Committee for the Purpose of Control and Supervision of Experiments on Animals Guidelines.” Thirty male Wistar rats (*n* = 30) were included in the current study and are divided into five groups of six rats in each group. Group I animals were administrated 0.9% normal saline for 14 days p.o., therefore served as control group. Group II served as toxic group, animals were administrated only INH-RIF (1:1) 100 mg/ kg b.wt for 14 days i.p. After one-hour of administration of INH-RIF (1:1) 100 mg/ kg to the animals of groups III, IV, and V, groups IV and V animals were administered with 100 mg/kg and 200 mg/kg b.wt CSP p.o, respectively whereas group III animals were administered with silymarin 10 mg/kg b.wt p.o. for 14 days. All rats were anaesthetized (ISOPLAN ^®^) and sacrificed after final treatment and blood samples were subsequently obtained for further experiments with dorsal venacava. The liver was extracted from the body of the animals for various investigations.

### 4.8. Biochemical Analysis

Blood samples collected from all groups were centrifuged and serum was assessed for various hepatic enzyme markers like aspartate aminotransferase (AST), alanine transaminase (ALT), alkaline phosphatase (ALP), and total protein TP by following reagent kits manuals.

### 4.9. Estimation of Catalase (CAT), Superoxide Dismutase (SOD) and Malondialdehyde (MDA)

The extracted liver was cleaned and homogenized in chilled buffered saline, the aliquot so obtained was used for the estimation of CAT [58], SOD [59], and MDA [60].

### 4.10. Proinflammatory Cytokine Analysis

Inflammatory mediators TNF-α and COX-2 were evaluated in serum by a commercial diagnostic kit.

### 4.11. Histological Evaluation

The histopathology study was evaluated by the procedure of Tahir et al. [61].

### 4.12. Statistical Analysis

Statistical Package for the Social Sciences (SPSS) Statistics Version 23 was used for the investigation of all the data. Differences among the groups were assessed using the variance analysis taken after by the Tukey-Kramer different comparison test and the least measure for measurable noteworthiness was set at *p* < 0.05 for all comparisons.

### 4.13. Statement of Ethical Approval

All procedures for using experimental animals were checked and proper permission was obtained from the Institutes animal ethics committee (Approval No: RAKMHSU-REC-7-2017-F-P).

## 5. Conclusions

The current study demonstrated that CSP significantly decreased the levels of hepatic enzyme and proinflammatory cytokines markers in the INH-RIF induced rats. The possible bioactive chemical compounds responsible for the hepatoprotective activity of CSP are rutin, morin, festin, and quercetin.

## Figures and Tables

**Table 1:** Chromatographic condition and characterization of chemical profiling of *Crocus sativus* L. petals (CSP) by LC–ESI-Q-TOF–MS.

Peak No.	Retention Time (min)	Molecular Formula	Theoretical (*m/z*)	Measured (*m/z*)	Compound Name
1	7.241	C_25_H_37_NO_5_	431.2722	432.2796	Hydroxysalmeterol
2	7.417	C_15_H_10_O_7_	302.0417	303.0490	Morin
3	7.704	C_21_H_20_O_11_	448.0995	449.1068	Quercetin
4	7.903	C_27_H_30_O_16_	610.1523	611.1595	Rutin
5	8.107	C_30_H_44_O_3_	452.335	454.3459	1alpha,25-dihydroxy-26,27-dimethyl-20,21,22,22,23,23-hexadehydro-24ahomovitaminD3
6	8.499	C_15_H_10_O_6_	286.047	287.0543	Fisetin
7	12.887	C_13_H_20_O_3_	224.1408	225.1481	Methyl jasmonate
8	13.477	C_10_H_8_O_3_	176.0473	177.0546	10-Hydroxy-8E-Decene-2,4,6-triynoic acid
9	13.526	C_17_H_26_O_5_	310.1768	311.1841	Methyl 8-[2-(2-formyl-vinyl)-3-hydroxy-5-*oxo*-cyclopentyl]-octanoate
10	15.085	C_12_H_25_NO	200.2003	199.193	Odecanamide

**Table 2:** Total phenolic content and flavonoids content of CSP.

Methods	CSP	R2
Total phenolic content (mg GAEa/g of extract)	89.63 ± 0.99	0.992
Total flavonoids content (μg QEb/g of extract)	65 ± 1.09	0.989

^a^ Total phenolics content is expressed in terms of gallic acid equivalent (μg of GAE/g); ^b^ Total flavonoids content is expressed in terms of quercetin equivalent (μg of QE/g). Values are expressed as mean ± standard deviation (*n* = 3).

**Table 3:** Antioxidant activity of CSP.

	CSP IC_50_ (μg/mL)	*R* ^2^	BHA IC_50_ (μg/mL)	*R* ^2^
DPPH Assay	99.53 ± 0.63	0.992	65.46 ± 1.22	0.901
ABTS Assay	116.63 ± 1.93	0.990	87.42 ± 0.990	0.989

All values are expressed as mean ± standard deviation (*n* = 3).

**Table 4:** Concentration of elements in CSP using inductively coupled plasma-optical emission spectrometry (ICP OES).

S. No	Name of the Element	Concentration (mg/Kg)
1.	Silver	0.2
2.	Aluminum	555
3.	Arsenic	0.2
4.	Boron	17.2
5.	Barium	3.9
6.	Beryllium	<0.1
7.	Calcium	4584
8.	Cadmium	<0.1
9.	Cobalt	0.2
10.	Chromium	1.6
11.	Copper	9.6
12.	Iron	1192
13.	Potassium	22,256
14.	Magnesium	1751
15.	Manganese	33.9
16.	Molybdenum	0.6
17.	Sodium	211
18.	Nickel	3.4
19.	Phosphorous	3569
20.	Lead	0.7
21.	Tin	0.1
22.	Selenium	0.1
23.	Strontium	9.1
24.	Vanadium	1.2
25.	Zinc	60.2

**Table 5:** Effects of the CSP on hepatic enzyme markers.

Groups	ALT (IUL^−1^)	AST (IUL^−1^)	ALP (IUL^−1^)	TP (g/dL)
Group I	62.23 ± 5.32	87.21 ± 5.23	100.43 ± 5.98	9.34 ± 0.21
Group II	143.90 ± 3.54 ^***^	302.43 ± 4.32 ^***^	278.21 ± 3.65 ^***^	3.18 ± 0.53 ^***^
Group III	90.98 ± 2.98 ^###^	95.01 ± 8.93 ^###^	138.32 ± 2.43 ^###^	7.02 ± 0.39 ^###^
Group IV	132.76 ± 4.34 ^##^	289.83 ± 8.93 ^#^	193.43 ± 3.64 ^##^	3.98 ± 0.53 ^#^
Group V	99.21 ± 7.03 ^##^	145.83 ± 6.83 ^###^	143.29 ± 4.83 ^###^	5.08 ± 0.98 ^##^

Annotation: *** *p* < 0.001 vs control, ^#^ *p* < 0.05, ^##^ *p* < 0.01, ^###^ *p* < 0.001 vs group II.

**Table 6:** Chromatographic condition (gradient system).

Time (min)	Function	Parameter
2.00	Change Solvent Composition	Solvent composition A: 95.00% B: 5.00%
2.00	Change Flow	Flow: 0.2 mL/min
2.00	Change Max. Pressure Limit	Max. Pressure Limit: 1200.00 bar
15.00	Change Solvent Composition	Solvent composition A: 5.00% B: 95.00%
15.00	Change Flow	Flow: 0.2 mL/min
24.00	Change Solvent Composition	Solvent composition A: 5.00% B: 95.00%
24.00	Change Flow	Flow: 0.2 mL/min
25.00	Change Solvent Composition	Solvent composition A: 95.00% B: 5.00%
25.00	Change Flow	Flow: 0.2 mL/min
30.00	Change Solvent Composition	Solvent composition A: 95.00% B: 5.00%

## Abbreviations

ABTS	(2,2′-azino-*bis*(3-ethylbenzothiazoline-6-sulfonic acid)
ALP	Alkaline phosphatase
ALT	Alanine aminotransferase
AST	Aspartate aminotransferase
ATC	Antituberculosis
CAT	Catalase
COX-2	Cyclooxygenase
CS	*Crocus sativus* L.
CSP	Crocus sativus petals
DIH	Drug-induced hepatotoxicity
DPPH	2,2-diphenyl-1-picry1hydrazy1
EMB	Ethambutol
GAE/g	Gallic acid equivalent per gram
IC50	Half maximal inhibitory concentration
ICP-OES	Inductively coupled plasma—optical emission spectrometry
INH	Isoniazid
LC-ESI-Q-TOFMS	Liquid chromatography coupled with electrospray ionization-quadrupole-time of flight-mass spectrometry
MDA	Malondialdehyde
PZA	Pymzinaide
QE/g	Quercetin equivalent per gram
R2	Regression
RIF	Rifampicin
SOD	Superoxide dismutase
TB	Tuberculosis
TFC	Total flavonoid content
TNF-α	Tumor necrosis factor alpha
TP	Total protein
TPC	Total phenolic content
WHO	World Health Organization
